# Do ureteral stents improve clinical outcomes in renal transplantation? A systematic review and meta-analysis comparing stented and non-stented anastomosis techniques

**DOI:** 10.7717/peerj.20665

**Published:** 2026-01-29

**Authors:** Shengnan Yin, Xiaodong Hao, Xiaoping Cai, Xiaowei Wang, Chenyang Zhao, Yaxiong Li, Shuo Zheng

**Affiliations:** 1 Department of Urology, The First Hospital of Hebei Medical University, Shijiazhuang, Hebei, China; 2 Department of Urology, Tongji Hospital Tongji Medical College of Huazhong University of Science and Technology, Wuhan, Hubei, China; 3 Department of Urology, Qinghe People Hospital, Qinghe, Hebei, China; 4 Department of Neurosurgery, The First Hospital of Hebei Medical University, Shijiazhuang, Hebei, China

**Keywords:** Renal transplantation, Ureteral stent, Urine leakage, Ureteral obstruction, Urinary tract infection

## Abstract

**Background:**

Urological complications following renal transplantation (RT) remain a significant clinical challenge. The role of ureteral stents in mitigating these complications is a subject of ongoing debate. This study aimed to assess whether ureteral stents improve clinical outcomes in RT, comparing stented and non-stented anastomosis techniques.

**Methods:**

An extensive search was conducted in PubMed, Embase, Cochrane Central Register of Controlled Trials, and the Chinese Biomedical Literature Service System from inception to November 26, 2025, following the PRISMA and AMSTAR standards. This study was registered with PROSPERO (CRD42024557423). The primary outcomes were urological mechanical complications (UMCs) and urinary tract infections (UTIs), whereas the secondary outcomes included hematuria, graft rejection, renal function, cost-effectiveness, stent-related complications, and quality of life (QOL). The Mantel-Haenszel test was used to calculate pooled risk ratios (RRs) and 95% confidence intervals (CIs) for the outcomes.

**Results:**

Sixteen RCTs involving 2,486 patients met the inclusion criteria. Meta-analysis revealed that the stent group had a significantly lower incidence of urine leakage (RR = 0.25, 95% CI [0.13–0.47]) and ureteral obstruction or stricture (RR = 0.42, 95% CI [0.25–0.71]) compared to the non-stent group. However, the incidence of UTIs was higher in the stent group (RR = 1.41, 95% CI [1.08–1.84]). No significant differences were observed in hematuria, graft rejection, or renal function between groups.

**Conclusions:**

Routine ureteral stent placement in RT significantly reduces the incidence of UMCs despite potentially increasing the risk of UTIs. This balance between benefits and risks supports the continued use of ureteral stents in RT, pending further high-quality studies.

## Introduction

Urological mechanical complications (UMCs) following renal transplantation (RT), including urine leakage, ureteral obstruction or stricture, significantly impact patient outcomes, quality of life (QOL), graft survival, cost-effectiveness, and length of hospital stay (Kumar, Kumar & Bhandari, 1998; Ooms et al., 2020). The effectiveness of ureteral stents in preventing postoperative complications is well-established (Murthy et al., 2017). However, with advancements in surgical techniques, adoption of enhanced immunosuppressive regimens, and improvements in post-operative care, the incidence of these complications has decreased significantly (Zaki et al., 2013).

According to a Cochrane review conducted by Wilson, Rix & Manas (2013), the implementation of routine prophylactic stenting has been proposed as a means to decrease the occurrence of UMCs. However, the majority of the studies encompassed in this review span the period from 1995 to 2000. Subsequently, an increasing number of randomized controlled trials (RCTs) have indicated that routine stent placement offers no advantage (Ooms et al., 2020; Murthy et al., 2017; Zaki et al., 2013; Osman et al., 2005; Majeed et al., 2022; Vinayak, Mukhopadhyay & Mittal, 2025). Therefore, the use of ureteral stents in RT remains controversial, particularly regarding their routine or selective application and the timing of removal (Doherty et al., 2021).

This systematic review and meta-analysis aims to assess whether ureteral stents improve clinical outcomes in RT, by comparing stented and non-stented anastomosis techniques.

## Materials & Methods

The systematic review and meta-analysis followed the PRISMA guidelines and AMSTAR standards (Page et al., 2021; Shea et al., 2017). This study was registered with PROSPERO (registration number CRD42024557423). The limited quantity of studies included prevented us from thoroughly analyzing all outcome indicators. To address this, we revised our search strategy across databases, refined some of the outcome measures, and excluded those indicators that did not exhibit clear relevance or significance.

### Search strategy

The search strategy was independently developed and executed by Shengnan Yin and Xiaoping Cai. Any disagreements were resolved by discussion and, when necessary, adjudicated by the third reviewer (Shuo Zheng). An optimally sensitive electronic search strategy was used to search PubMed, Embase, Cochrane Central Register of Controlled Trials (CENTRAL), and Chinese Biomedical Literature Service System (SinoMed). By November 26, 2025, we will conduct an exhaustive search for all relevant articles, imposing no limitations on country of origin or article type. Furthermore, the reference lists of all selected articles will undergo an independent screening process to uncover any supplementary studies that may have been overlooked during the initial search phase. The search terms used included: (Kidney transplantation, Renal transplantation, Nephrotransplantation, Kidney graft, Renal transplant, Renal graft, and Renal allograft), (Ureteral stent, Ureteric stent, Urinary stent, Ureteral stent, Ureteral splint, Urologic stent, Double-J stent, JJ Stent, Stent placement, Ureteral catheter, Ureteral drainage tube, and Ureteral implant). Boolean operators (OR and AND) were used to refine the search.

### Criteria for considering studies

***Inclusion criteria*:**

Type of study: RCTs;

The participants were renal transplant recipients who underwent the procedure with or without ureteral stent catheter placement during surgery.

No restrictions on the recipient’s race, nationality, age, or gender, and the source of the kidney, whether from a deceased or living donor, is not limited;

The method of ureterovesical anastomosis is not restricted;

Multi-organ combined transplantations, including the kidney, are also included.

***Exclusion criteria*:**

Patients with horseshoe kidneys and abnormal bladder function;

Patients with urinary diversion and lower urinary tract lesions;

Patients with a retention time of ureteral stent in the body of less than 3 days;

Renal autotransplantation patients;

Literature that does not report efficacy determination criteria or lacks detailed data.

### Outcomes

***Primary outcomes***

The primary outcomes were UMCs (urine leakage, ureteral obstruction, or stricture) and UTIs.

***Secondary outcomes***

Secondary outcomes included Hematuria, Graft rejection, renal function, Cost-effectiveness, Other stent-related complications (stone formation, stent breakage, migration and secondary obstruction), and QOL.

### Quality assessment

Xiaowei Wang and Yaxiong Li independently conducted a methodological quality evaluation of the included studies, utilizing the modified Jadad scale as the assessment tool (Zhao et al., 2023). The modified Jadad scale was applied to assess randomization, allocation concealment, and double-blinding methods, with a score of 2 awarded for those deemed appropriate, 1 for unclear methods, and 0 for inappropriate methods. Studies that described withdrawals or missing visits received a score of 1, while those without any such description received a score of 0. The cumulative score, with a maximum of 7, indicated the overall quality of the study; a total score of 4 or below was indicative of poor study quality.

### Data extraction and analysis

Literature screening was performed between 26 November 2025 and 28 November 2025; data extraction was conducted from 29 November 2025 to 1 December 2025. Data extraction was conducted independently by Xiaodong Hao and Shuo Zheng. They extracted information on the journal, authors, number of patients, study type, publication date, mean age, type of donor (living or deceased), ureteric implantation technique (extravesical, transvesical), urological complications (urine leakage, ureteral obstruction or stricture, UTIs, hematuria), stent removal time, indwelling catheter duration, graft rejection, renal function, cost-effectiveness, and QOL were extracted. Disagreements were resolved by engaging in discussions with an additional author (Chenyang Zhao).

Statistical analysis was conducted using Review Manager version 5.4. Statistical significance was defined as *p* < 0.05. Dichotomous variables were collected, and outcome measures were combined using a random-effects model when possible. The Mantel–Haenszel test was used to calculate pooled risk ratios (RRs) and 95% confidence intervals (CIs) for the outcomes. To evaluate whether the observed heterogeneity in results exceeded that expected by chance, the chi-squared (X^2^) test for heterogeneity and I^2^ statistic were utilized, and when the I^2^ value was less than 70%, the heterogeneity was acceptable. Publication bias was assessed utilizing inverted funnel plots. Participants who withdrew, dropped out were still accounted for in the intention-to-treat (ITT) analysis. Sensitivity analysis was performed to assess the reliability of the results when significant heterogeneity was observed.

## Results

### Study selection

**Figure 1:**
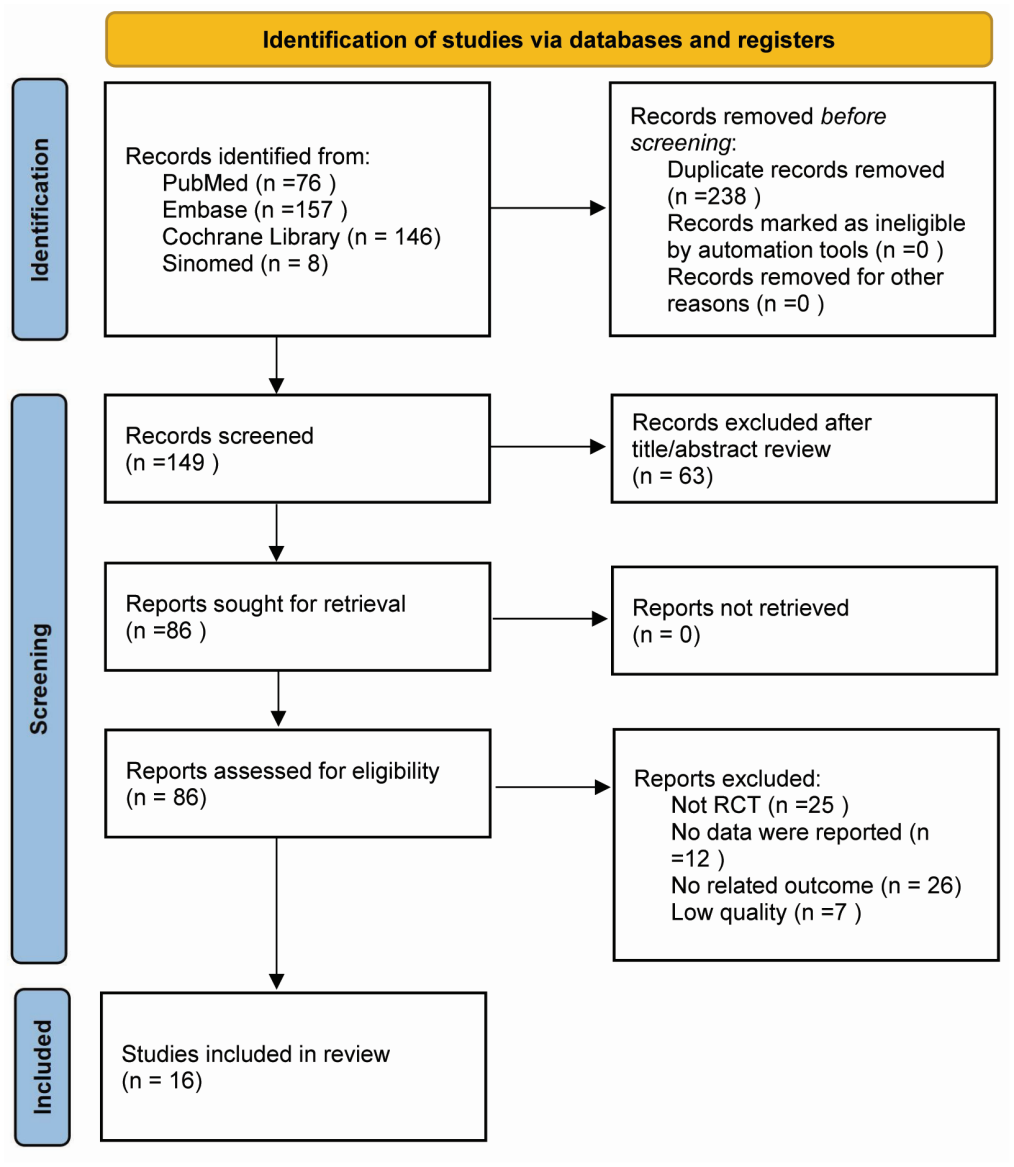
PRISMA diagram showing selection of articles for review.

The PRISMA diagram illustrates the outcomes of the literature search (Fig. 1). We initially identified 387 relevant studies using the electronic databases. After eliminating duplicates, 149 records remained in total. Screening of these records by title and abstract resulted in 86 full-text review studies. Sixteen studies, encompassing 2,486 patients, met the inclusion criteria and were included in the final review (Kumar, Kumar & Bhandari, 1998; Ooms et al., 2020; Murthy et al., 2017; Zaki et al., 2013; Osman et al., 2005; Majeed et al., 2022; Bassiri et al., 1995; Pleass et al., 1995; Benoit et al., 1996; Guleria et al., 1998; Dominguez et al., 2000; Moray et al., 2005; Tavakoli et al., 2007; Dong et al., 2002; Wang & Zhang, 2009; Vinayak, Mukhopadhyay & Mittal, 2025).

### Study characteristics and quality assessment

Table 1 outlines the baseline characteristics of the studies included in the meta-analysis. 1,265 and 1,221 patients were in the stent group and no-stent group, respectively. In these studies, 11 studies described kidney transplantation from living donors (Kumar, Kumar & Bhandari, 1998; Ooms et al., 2020; Murthy et al., 2017; Zaki et al., 2013; Osman et al., 2005; Majeed et al., 2022; Bassiri et al., 1995; Guleria et al., 1998; Moray et al., 2005; Dong et al., 2002; Vinayak, Mukhopadhyay & Mittal, 2025). Different stent types (length and caliber) were used in these studies. Most studies have adopted the Lich-Gregoire surgical technique. We found that the heterogeneities were due to donor source, stent length/caliber, stent removal time, indwelling catheter duration, and antibiotic regimen. Table 2 displays the bias risk assessment outcomes.

**Table 1:** Characteristics of the included studies.

**Study (yr)**	**Number of patients**	**Number stented/no stented**	**Donor source**	**Stent length/ caliber**	**Stent removal time**	**Indwelling catheter duration**	**Antibiotic regimen**	**Type of surgery**	**The definition of UTI**	**Outcomes**	**Conclusions**
Bassiri 1995	72	35/37	L	16 cm/NR	6–8 weeks	NR	NR	L-G	NR	①②③⑤	Recommended
Pleass 1995	300	150/150	L/D	12 cm/7Fr	3 months	5 days	Cotrimoxazole	L-G	Clinically (fever/symptoms) and confirmed on microscopy	①②③⑤	Recommended
Benoit 1996	194	97/97	L/D	16 cm/7Fr	1 months	3 days	Cotrimoxazole	L-G and L-P	Positive culture >10^5^/ml, and acute pyelonephritis	①②③④⑦⑧	Significantly decreases
Guleria 1998	108	54/54	L	24 cm/NR	14–21 days	NR	Cephalexin	L-G and L-P	Positive culture	①②③	Not be recommended
Kumar 1998	100	57/43	L	16 cm/6Fr	4 weeks	5 days	Cotrimoxazole	L-G	Positive culture	①②③⑤⑥⑧	Recommended
Dominguez 2000	280	143/137	L/D	12 cm/6Fr	7–10 days	48 hr	Cotrimoxazole	L-G	Positive culture >10^5^/ml, with fever or urinary symptoms	①②③④	Unnecessary
Dong 2002	230	114/116	L	15 cm/6Fr	3–4 weeks	5 days	Ofloxacin	L-G	Positive culture >10^5^/ml, with fever and/or urinary symptoms	①②③	Significantly decreases
Osman 2005	100	50/50	L	NR/5Fr	2 weeks	4–6 days	Ampicillin+treat based on urine culture	L-G	Positive culture	①②③⑤⑦⑧	Noninferiority
Moray 2005	42	21/21	L	NR	1 months	NR	NR	L-G	NR	①③④⑥⑦	Surgeon’s discretion
Tavakoli 2007	201	112/89	L/D	16 cm/6Fr	Mean 74.3 days	5 days	Coamxiclave+ Cotrimoxazole	L-G	Positive culture >10^5^/ml or urine >100 WBC /HP, with fever and/or urinary symptoms	①②③④⑥	Significantly decreases
Wang 2009	95	50/45	D	NR/6Fr	10–14 days	1 week	Treat based on urine culture	L-G	Positive culture with urinary symptoms	①②③	Significantly decreases
Zaki 2013	300	150/150	L	NR	3–4 weeks	NR	3rd generation cephalosporin	L-G	NR	①②③⑤	No difference
Murthy 2017	76	38/38	L	NR	4 weeks	3 days	NR	L-G	Positive culture	①②③⑦	Not be indicated
Ooms 2020	200	100/100	L	NR/7Fr single J stent	9 days	7 days	NR	L-G	Positive culture >10^5^/ml, treated with antibiotics.	①②③④⑤	Noninferiority
Majeed 2022	108	54/54	L	NR	NR	NR	NR	L-G	NR	①	Not mandatory
Vinayak 2025	80	40/40	L	16 cm/6Fr	3 weeks	5 days	NR	L-G	Positive culture	①②③	Not necessary

**Notes.**

UTI urinary tract infection L living L/D living/deceased NR no report L-G Lich-Gregoire technique L-P Leadbetter-Politano technique WBC/HP White Blood Cell/High Power Field ① urine leakage ② ureteral obstruction or stricture ③ UTIs ④ graft rejection ⑤ hematuria ⑥ cost ⑦ renal function ⑧ stent related problems (stent migration, breakage, stone formation, and secondary obstruction) (Kumar, Kumar & Bhandari, 1998; Ooms et al., 2020; Murthy et al., 2017; Zaki et al., 2013; Osman et al., 2005; Bassiri et al., 1995; Pleass et al., 1995; Benoit et al., 1996; Guleria et al., 1998; Dominguez et al., 2000; Tavakoli et al., 2007; Dong et al., 2002; Wang & Zhang, 2009; Vinayak, Mukhopadhyay & Mittal, 2025; Majeed et al., 2022; Moray et al., 2005)

**Table 2:** Risk of bias in the included studies (modified Jadad scale) (Kumar, Kumar & Bhandari, 1998; Ooms et al., 2020; Murthy et al., 2017; Zaki et al., 2013; Osman et al., 2005; Bassiri et al., 1995; Pleass et al., 1995; Benoit et al., 1996; Guleria et al., 1998; Dominguez et al., 2000; Tavakoli et al., 2007; Dong et al., 2002; Wang & Zhang, 2009; Vinayak, Mukhopadhyay & Mittal, 2025; Moray et al., 2005; Majeed et al., 2022).

**Study (yr)**	**Random sequence generation**	**Allocation concealment**	**Blinding**	**Withdrawals and dropouts**	**Jadad score**
**Bassiri 1995**	1	0	0	1	2
**Pleass 1995**	2	1	0	1	4
**Benoit 1996**	1	0	0	1	2
**Guleria 1998**	1	2	0	1	4
**Kumar 1998**	2	1	0	1	4
**Dominguez 2000**	2	1	0	1	4
**Dong 2002**	1	1	0	0	2
**Osman 2005**	2	1	0	1	4
**Moray 2005**	1	1	0	0	2
**Tavakoli 2007**	1	2	0	1	4
**Wang 2009**	1	1	0	1	3
**Zaki 2013**	1	1	0	1	3
**Murthy 2017**	2	2	0	1	5
**Ooms 2020**	2	2	0	1	5
**Majeed 2022**	1	1	0	0	2
**Vinayak 2025**	2	0	0	1	3

### Urine leakage and Ureteral obstruction or stricture

**Figure 2:**
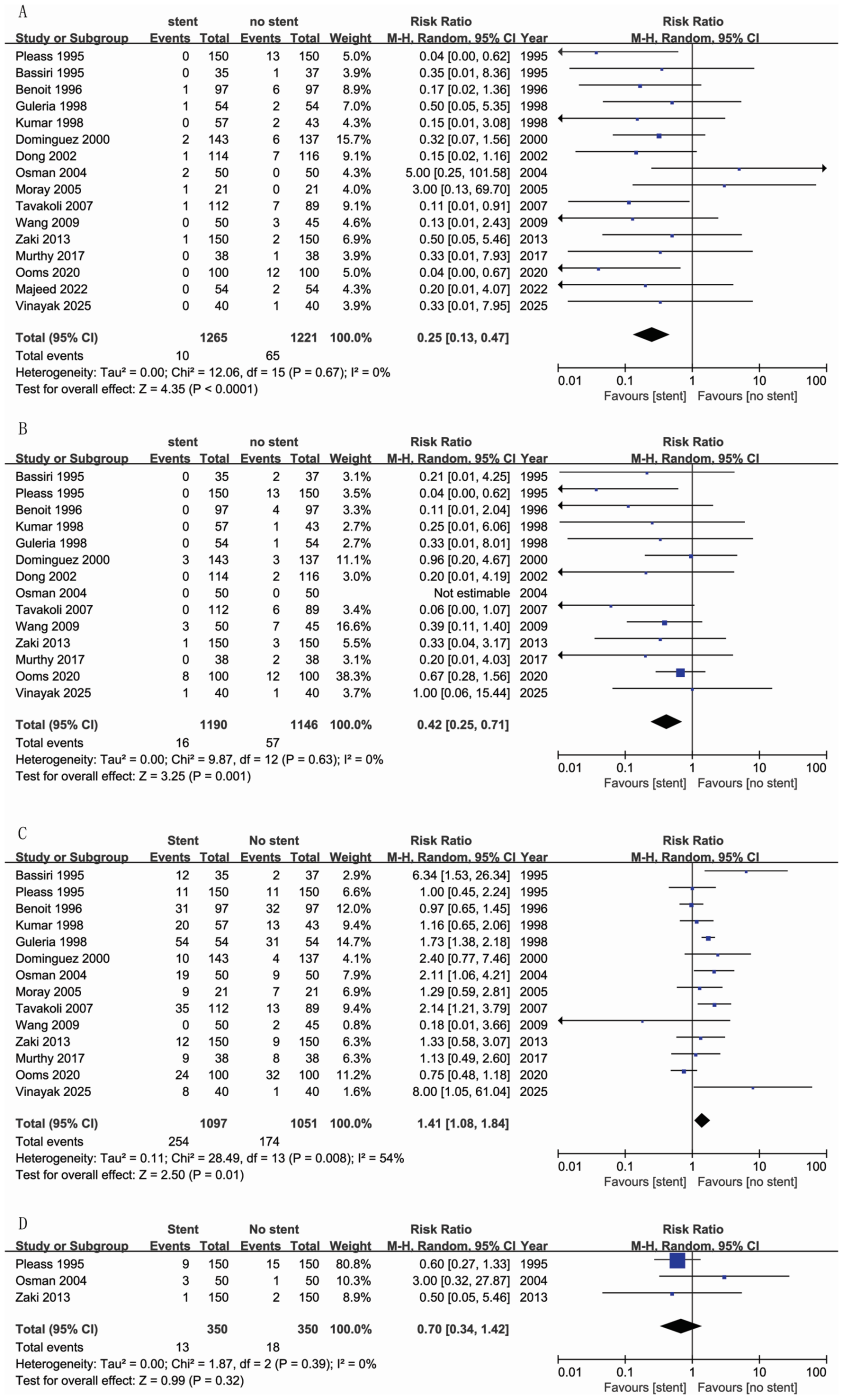
Forest plot for urine leakage (A), ureteral obstruction or stricture (B), UTI (C), and hematuria (D) (Kumar, Kumar & Bhandari, 1998; Ooms et al., 2020; Murthy et al., 2017; Zaki et al., 2013; Osman et al., 2005; Bassiri et al., 1995; Pleass et al., 1995; Benoit et al., 1996; Guleria et al., 1998; Dominguez et al., 2000; Tavakoli et al., 2007; Dong et al., 2002; Wang & Zhang, 2009; Vinayak, Mukhopadhyay & Mittal, 2025; Majeed et al., 2022).

Sixteen studies (2,486 patients) reported urine leakage (Kumar, Kumar & Bhandari, 1998; Ooms et al., 2020; Murthy et al., 2017; Zaki et al., 2013; Osman et al., 2005; Majeed et al., 2022; Bassiri et al., 1995; Pleass et al., 1995; Benoit et al., 1996; Guleria et al., 1998; Dominguez et al., 2000; Moray et al., 2005; Tavakoli et al., 2007; Dong et al., 2002; Wang & Zhang, 2009; Vinayak, Mukhopadhyay & Mittal, 2025). The meta-analysis revealed a significantly lower incidence rate of urine leakage in the stent group compared to the no-stent group (RR = 0.25, 95% CI [0.13–0.47]; I^2^ = 0%) (Fig. 2A).

Ureteral obstruction or stricture was recorded in 14 studies (Kumar, Kumar & Bhandari, 1998; Ooms et al., 2020; Murthy et al., 2017; Zaki et al., 2013; Osman et al., 2005; Bassiri et al., 1995; Pleass et al., 1995; Benoit et al., 1996; Guleria et al., 1998; Dominguez et al., 2000; Tavakoli et al., 2007; Dong et al., 2002; Wang & Zhang, 2009; Vinayak, Mukhopadhyay & Mittal, 2025), involving 2,336 patients. Pooled analysis showed a significantly reduced incidence of ureteral obstruction or stricture in the stent group (RR = 0.42, 95% CI [0.25–0.71]; I^2^ = 0%) (Fig. 2B).

### UTI

Considerable heterogeneity existed across studies in the definitions, reported morbidity and relative incidence of UTI. UTI was defined according to the original criteria adopted by each trial (Table 1). Fourteen studies were included in this meta-analysis (Kumar, Kumar & Bhandari, 1998; Ooms et al., 2020; Murthy et al., 2017; Zaki et al., 2013; Osman et al., 2005; Bassiri et al., 1995; Pleass et al., 1995; Benoit et al., 1996; Guleria et al., 1998; Dominguez et al., 2000; Moray et al., 2005; Tavakoli et al., 2007; Wang & Zhang, 2009; Vinayak, Mukhopadhyay & Mittal, 2025). The incidence of UTI in the stent group was 23.15% compared to 16.56% in the no-stent group, with a statistically significant difference (RR = 1.41, 95% CI [1.08–1.84]; I^2^ = 54%) (Fig. 2C). Different studies have defined UTI as Positive culture of >105 per ml or urine greater than 100 WBC/HP, with fever and/or urinary symptoms (Pleass et al., 1995; Benoit et al., 1996; Dominguez et al., 2000; Tavakoli et al., 2007; Dong et al., 2002; Wang & Zhang, 2009). Some studies define UTI as a positive culture (Kumar, Kumar & Bhandari, 1998; Ooms et al., 2020; Murthy et al., 2017; Osman et al., 2005; Guleria et al., 1998; Vinayak, Mukhopadhyay & Mittal, 2025). The choice of postoperative antibiotics is different; they could be third-generation cephalosporins (Zaki et al., 2013), ofloxacin (Dong et al., 2002), cotrimoxazole (Kumar, Kumar & Bhandari, 1998; Pleass et al., 1995; Benoit et al., 1996; Dominguez et al., 2000; Tavakoli et al., 2007), cephalexin (Guleria et al., 1998), or administered based on culture results (Osman et al., 2005; Wang & Zhang, 2009) (Table 1).

### Hematuria

Three studies that required the management of hematuria were included in the meta-analysis (Zaki et al., 2013; Osman et al., 2005; Pleass et al., 1995). No significant difference was found between the stent and no-stent groups (RR = 0.70, 95% CI [0.34–1.42]; I^2^ = 0%) (Fig. 2D). However, Ooms et al. (2020) defined hematuria as macroscopic hematuria during hospital admission. They reported that the stent group experienced a higher rate of hematuria than the no-stent group within one month (76% *vs.* 50%).

### Graft rejection

Only four studies, involving 637 patients reported graft rejection (Ooms et al., 2020; Benoit et al., 1996; Moray et al., 2005; Tavakoli et al., 2007). Notably, only one study confirmed rejection through renal biopsy (Moray et al., 2005), conducted when a patient’s creatinine level stayed elevated for three days post-transplantation. Among the biopsies, four from the stent group and three from the no-stent group indicated acute rejection. Tavakoli et al. (2007) reported that acute rejection occurred more frequently in the stent group than in the no-stent group (52.68% *vs.* 31.46%). Similarly, in another study, a higher proportion of patients required treatment for graft rejection in the stent group (36%) than in the no-stent group (21%). However, the Benoit study reported a rejection incidence of 38.1% in the stent group *versus* 42.3% in the no-stent group (Benoit et al., 1996).

### Renal function

Two studies indicated that the changes in serum creatinine levels were not statistically significant between the stent and no-stent groups (Murthy et al., 2017; Osman et al., 2005). Osman et al. (2005) noted that the average serum creatinine levels at discharge were 1.2 ± 0.3 mg% in the stent group and 1.2 ± 0.4 mg% in the no stent group (*p* = 0.2). Similarly, Murthy et al. observed that the mean serum creatinine levels were 1.308 in the stent group and 1.37 in the no-stent group (*p* = 0.609).

### Other stent related complications

Three studies (Kumar, Kumar & Bhandari, 1998; Osman et al., 2005; Tavakoli et al., 2007), reported no stent-related problems, such as stone formation, stent breakage, migration and secondary obstruction. Fewer stent-related complications occurred (one stent migration and two stent breakages in Benoit’s study; one stent migration in Zaki’s study) (Zaki et al., 2013; Benoit et al., 1996).

### Cost

Owing to the varying economic standards and price levels among different countries and regions, cost comparisons often lack consistency. One study reported that the mean cost per patient as £755 for the stent group, and £906 for the no-stent group (Tavakoli et al., 2007). The primary expense was attributed to the placement and subsequent removal of the stent in the stent group. Conversely, in the no-stent group, all additional costs were exclusively linked to managing the arising complications. Similarly, Kumar, Kumar & Bhandari (1998) noted that the additional cost related to the use of a stent was an RS 1,600 per patient. However, this additional expense was significantly offset by the costs incurred in managing urological complications in patients without stents, which amounted to RS 700 per patient.

### QOL

Only one study has conducted a quality of life (QOL) survey (Ooms et al., 2020). The Euro-Qol-5D and Short Form 36 (SF-36) questionnaires, were administered before surgery and at various postoperative intervals (2 and 6 weeks, and 3, 6, 9, and 12 months). The study revealed that, while the no-stent group exhibited superior Euro-Qol-5D scores at 2 and 6 weeks postoperatively (*p* = 0.030 and *p* = 0.037, respectively), but both groups showed similar quality of life over time according to SF-36 and Euro-Qol-5D scores.

### Sensitivity analysis and publication bias

**Figure 3:**
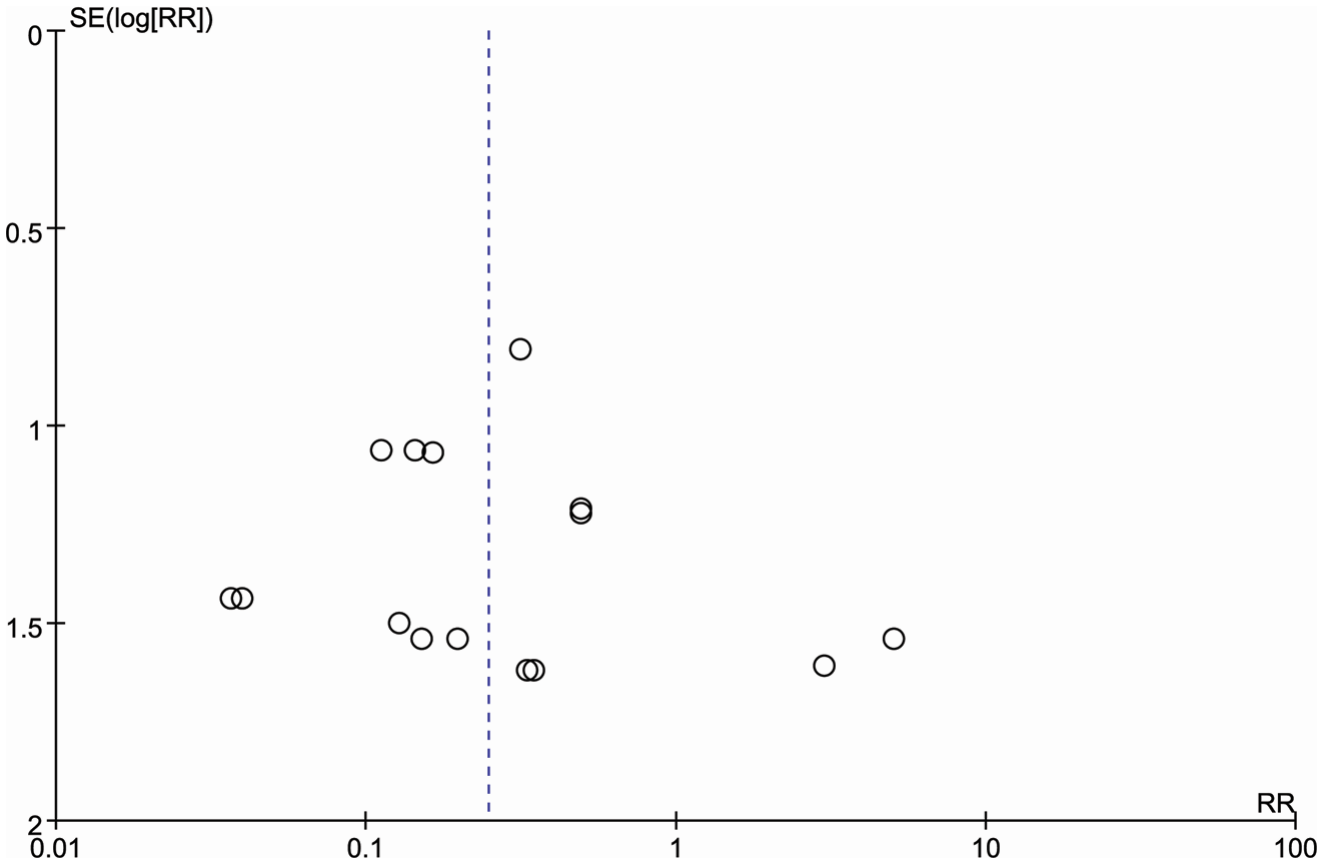
Funnel plot of urine leakage.

Sensitivity analysis was conducted by switching from a random-effects model to a fixed-effects model for data synthesis. The outcomes of the fixed-effects model mirrored those obtained from the random-effects model, demonstrating robustness in the findings. Sensitivity analysis demonstrated the stability of the results. Publication bias was evaluated with a funnel plot of urinary leaks. The funnel plot showed a predominantly symmetrical distribution with most points concentrated in the upper section. The analysis in this study appears stable and credible, with minimal risk of publication bias (Fig. 3).

## Discussion

Ureteral stents can effectively prevent complications in the urinary system. Although advancements in surgical techniques over the past 30 years have halved the incidence of urinary system complications, they remain common after RT. These complications include urine leakage, ureteral obstruction, or stricture (Kırnap et al., 2019), which are usually caused by damage to the ureteral blood supply during donor nephrectomy due to ureteral vascular injury and obstruction of the anastomotic site caused by ureteral inflammation or edema. The placement of ureteral stents facilitates urine flow from the kidney to the bladder, reduces intraureteral pressure, and prevents ischemia-related necrosis of the distal ureter and urinary leakage (Doherty et al., 2021; Lee, Katz & Shah, 2021).

However, the use of ureteral stents in RT has always been a topic of great interest. While early meta-analyses tended to support the routine placement of stents to prevent postoperative complications, six recent RCTs have presented a contrasting view, suggesting that for many patients, stent placement may not be necessary and could even pose additional risks (Ooms et al., 2020; Murthy et al., 2017; Zaki et al., 2013; Osman et al., 2005; Majeed et al., 2022; Vinayak, Mukhopadhyay & Mittal, 2025). This controversy highlights the inconsistencies and complexities of current research, underscoring the need for a updated systematic review and meta-analysis.

Our analysis shows that ureteral stents significantly reduce the risk of UMCs. This is consistent with early research findings, supporting the use of ureteral stents in certain circumstances. However, we also found that the use of ureteral stents is linked to a higher risk of UTIs. The relevant mechanism may be attributed to the ureteral stent acting as a foreign body, facilitating rapid formation of a microbial biofilm on its surface. During bladder detrusor contraction, a small volume of urine-carrying bacteria can reflux into the renal pelvis, thereby inducing pyelonephritis (Visser et al., 2019). Judicious application of prophylactic antibiotics resulted in no statistically significant difference in UTI incidence between the stented and non-stented cohorts (Wilson, Rix & Manas, 2013; Suárez Fernández et al., 2021). Different antibiotics were used for perioperative treatment in our study (Table 1). Studies have shown that Prophylactic use of trimethoprim or sulfamethoxazole, early targeted antibiotic therapy for patients with positive urine cultures, early stent removal, and regular follow-up after stent removal are effective strategies for reducing the incidence of upper urinary tract infections after transplantation (Lee et al., 2019).

Stent duration is a critical variable that must balance the reduction of UMCs against the increased risk of UTIs, particularly with longer indwelling durations. Concurrently, significant advances in ureteral stent technology have been achieved through the exploration and development of novel bioactive coating materials (Wen et al., 2024). The material and design of a stent significantly influence its patency duration and complication profile. Although our included studies did not directly analyze this aspect, future clinical trials are expected to provide further supporting evidence. These findings indicate a need to balance the prevention of UMCs with the reduction of UTI risk.

Among the 16 trials included in our analysis, 11 involved living donors, one involved exclusively deceased donors, and four involved both living and deceased donors (Table 1). This significant imbalance with only one study involving purely deceased donors precluded meaningful subgroup analysis, and thus no comparative analysis between living and deceased donor transplants was performed. Notably, six of these studies suggested that stent placement may be unnecessary or even associated with additional risks, though all of them involved only living donors (Ooms et al., 2020; Murthy et al., 2017; Zaki et al., 2013; Osman et al., 2005; Majeed et al., 2022; Vinayak, Mukhopadhyay & Mittal, 2025). Kidneys from living donors generally exhibit better tissue compatibility and shorter cold ischemia times, which may help reduce the risk of certain complications (Simpkins et al., 2007; van de Laar et al., 2022). However, other factors such as surgical technique, health status of the donor and recipient, and postoperative management also play important roles (Baker et al., 2017). Therefore, whether the source of the kidney transplant (living or deceased donor) affects the necessity of ureteral stenting warrants additional investigation.

The incidence of hematuria ranges from 1–34% (Kayler et al., 2010). Most studies utilized the Lich-Gregoire technique, which results in the lowest incidence of hematuria. A meta-analysis suggested that the incidence of hematuria requiring intervention was 3.71% in the stent group and 5.14% in the no-stent group, neither of which was considered high. Meticulous hemostasis during reimplantation results in minimal bleeding (Kayler et al., 2010; Alberts et al., 2014; Secin et al., 2002).

However, the impact of stent placement on the incidence of renal allograft rejection remains unclear. Owing to the instability observed in the sensitivity analyses, we did not perform a meta-analysis to assess the effect. Moray et al. (2005) proposed that ureteral stasis could potentially lead to tubuloepithelial damage and impede the reduction of creatinine levels. Based on this theory, stents may reduce the incidence of acute rejection.

Based on the above, our study highlights the need for individualized assessment in stent placement, considering patient conditions and risks to avoid unnecessary interventions. Strategies include: (1) Individualized decision-making: Based on patient specifics and clinical judgment, decide on stent placement. High-risk patients may benefit from stents, while low-risk ones may not require them; (2) Close postoperative monitoring: Regardless of stent use, closely monitor patients to detect and manage complications promptly; (3) Further research: Explore optimal stent use in diverse populations, factors affecting outcomes, and stent impact on graft survival, costs, and QoL.

Nonetheless, this study has limitations, such as the variable quality of the included studies and insufficient data for some outcome indicators. And minimally invasive kidney transplant is gaining popularity and the use of more sophisticated technologies could change the use of ureteral stent even for kidney transplant. For instance, robot-assisted kidney transplantation (RAKT) has become a valid alternative to open kidney transplantation (OKT) (Territo et al., 2025). A study comparing RAKT and OKT in deceased donors found comparable short-term and long-term functional outcomes. However, RAKT showed advantages in reducing rewarming and vascular anastomosis times, while OKT had shorter median days for drain and JJ stent removal (Afferi et al., 2025). Additionally, the transplant team’s experience is a potentially significant determinant of surgical outcomes. While difficult to measure precisely, it is reasonable to infer that more experienced teams may achieve better results, manage complications more adeptly, and make better decisions about stent placement. Therefore, future research should further improve the study design and data collection methods to enhance the reliability and generalizability of the results.

## Conclusions

In conclusion, by integrating the latest evidence, this systematic review and meta-analysis indicates that routine ureteral stent placement in renal transplantation remains a reasonable choice, which improves clinical outcomes, such as urinary leakage, ureteral obstruction, or stricture. However, we also recognize that controversy still exists in this field, and future high-quality studies are needed to confirm this outcome.

##  Supplemental Information

10.7717/peerj.20665/supp-1 Supplemental Information 1

10.7717/peerj.20665/supp-2 Supplemental Information 2 Search Strategy

10.7717/peerj.20665/supp-3 Supplemental Information 3 PRISMA checklist

10.7717/peerj.20665/supp-4 Supplemental Information 4 Raw data

## Additional Information and Declarations

### Data Availability

The following information was supplied regarding data availability: The raw measurements are available in the Supplementary File. This is a Systematic review/Meta-analysis.
