# Supplementary material for: Do ureteral stents improve clinical outcomes in renal transplantation? A systematic review and meta-analysis comparing stented and non-stented anastomosis techniques
Source: PeerJ. 2026 Jan 29;14:e20665. doi: 10.7717/peerj.20665 (PMC12861130; doi:10.7717/peerj.20665)
Supplement: Supplemental Information 2 [file peerj-14-20665-s002.docx]

**Search Strategy**

**PubMed**

#1 "kidney transplantation"[MeSH Terms]

#2 "renal transplantation" OR "Nephrotransplantation" OR "Kidney graft" OR "Renal transplant" OR "Renal graft" OR "Renal allograft"

#3 #1 OR #2

#4 "Ureteral Stent" OR "Ureteric Stent" OR "Urinary Stent" OR "Ureter Stent" OR "Ureteral Splint" OR "Urologic Stent" OR "Double-J Stent" OR "JJ Stent" OR "Stent placement" OR "Ureteral catheter" OR "Ureteral Drainage Tube" OR "Ureteral Implant"

#5 #3 AND #4

**Embase**

#1 'kidney transplantation'/exp OR 'renal transplantation'/exp OR nephrotransplantation OR 'kidney graft'/exp OR 'renal transplant'/exp OR 'renal graft'/exp OR 'renal allograft'/exp

#2 'ureter stent'/exp OR 'ureteral stent'/exp OR 'ureteric stent'/exp OR 'urinary stent'/exp OR 'ureteral splint' OR 'urologic stent'/exp OR 'double-j stent'/exp OR 'jj stent'/exp OR 'stent placement'/exp OR 'ureteral catheter'/exp OR 'ureteral drainage tube' OR 'ureteral implant'

#3 #1 AND #2

**Cochrane Library**

#1 MeSH descriptor: [Kidney Transplantation] explode all trees

#2 (Renal graft):ti,ab,kw OR (Renal allograft):ti,ab,kw OR (Nephrotransplantation):ti,ab,kw OR (Kidney graft):ti,ab,kw OR (Renal transplant):ti,ab,kw in Trials (Word variations have been searched)

#3 MeSH descriptor: [Kidney Transplantation] explode all trees

#4 #1or#2or#3 in Trials

#5 (Ureteral Stent):ti,ab,kw OR (Ureteric Stent):ti,ab,kw OR (Urinary Stent):ti,ab,kw OR (Ureteral Splint):ti,ab,kw AND (Ureter Stent):ti,ab,kw in Trials (Word variations have been searched)

#6 (Urologic Stent):ti,ab,kw OR (Double-J Stent):ti,ab,kw OR (JJ Stent):ti,ab,kw OR (Stent placement):ti,ab,kw OR (Ureteral catheter):ti,ab,kw in Trials (Word variations have been searched)

#7 (Ureteral Drainage Tube):ti,ab,kw OR (Ureteral Implant):ti,ab,kw in Trials (Word variations have been searched)

#8 #5OR#6OR#7 in Trials

#9 #4AND#8 in Trials

**Chinese Biomedical Literature Service System (SinoMed)**

#1 "肾移植"[加权:扩展] OR "移植肾"[常用字段:智能]

#2 "输尿管支架"[关键词:智能] OR "支架"[常用字段:智能] OR "双J管"[常用字段:智能] OR "双J"[常用字段:智能]

#3 #1 AND #2
